# A New Diagnostic Way for Behcet's Disease: Skin Prick with Self-Saliva

**DOI:** 10.1155/2014/581468

**Published:** 2014-01-23

**Authors:** Fumio Kaneko, Ari Togashi, Erika Nomura, Koichiro Nakamura

**Affiliations:** ^1^Institute of Dermato-Immunology and -Allergy, Southern TOHOKU Research Institute for Neuroscience, 7-115 Yatsuyamada, Koriyama, Fukushima 963-8563, Japan; ^2^Department of Dermatology, Saitama Medical University, 38 Hongo, Moroyama Iruma-gun, Saitama 350-0495, Japan

## Abstract

Behcet's disease (BD) is a mysterious multisystemic disorder characterized by recurrent involvement of mucocutaneous (including recurrent aphthous stomatitis; RAS), ocular, intestinal, vascular, and/or nervous system organs. Previously, the positivity of “pathergy test”, which is one of the diagnostic examinations, was reported to be related to the possession of HLA-B51 gene in BD patients, even though the positivity is low and different from the countries. Here, instead of the ordinal pathergy test, we would like to propose the prick with self-saliva as a new diagnostic way for patients with RAS of BD based on the genetic intrinsic factors including HLA-B51 and extrinsic triggering factors. BD patients are considered to acquire the hypersensitivity against oral *streptococci* through the innate immune mechanism in the oral cavity. *Bes-1* gene and 65 kD of heat shock protein (HSP-65) derived from oral *S. sanguinis* are supposed to play important roles as extrinsic factors in BD pathogenesis. Although the prick positivity was not related to the possession of HLA-B51 gene, the method is suggested to be a significant way for BD diagnosis. The results also suggest that BD symptoms are due to the vascular immune responses by monocytes expressed oral streptococcal agents of the patients.

## 1. Introduction

Behcet's disease (BD) [1] is a chronic multisystematic inflammatory disorder characterized by the recurrent involvement of mucocutaneous [oral and genital ulceration, erythema nodosum (EN)-like eruption, acne-like eruption, etc.], ocular, vascular, digestive, and/or nervous system organs. Although the actual etiology of BD is still unclear, the pathogenesis has been generally clearer by the etiological research based on the genetic intrinsic factors and immunological reactions to the extrinsic triggering factors in an environmental agent [2–14]. As one of the triggering factors, the oral unhygienic condition may be suspected, because periodontitis, decayed teeth, chronic tonsillitis, and so forth are frequently noted in the oral cavity of BD patients [9, 10]. The infectious triggering factors are suspected to be many organisms including streptococci, herpes simplex viruses (HSVs), *Saccharomyces fermentans, Borrelia burgdorferi, Helicobacter pylori, Escherichia coli, Staphylococcus aureus, Mycoplasma fermentans,* and *mycobacterium* [11]. The proportion of *Streptococcus sanguinis (S. sanguinis),* which was previously recognized as *species* of the *genus Streptococcus* named “*S. sanguis*,” was significantly high in the oral bacterial flora of BD patients in comparison with those of healthy controls [12–14]. Most of the patients tend to acquire hypersensitivity against *streptococci* in their oral bacterial flora, as previously demonstrated that much stronger cutaneous reactions were seen by the prick with streptococcal antigen than those by “Pathergy test” [8, 9, 15, 16]. Non-BD patients with recurrent aphthous stomatitis (non-BD-RAS) were also having the hyperreactivity as reported by Graykowski et al. in the 1960s [17]. In vitro system, inflammatory cytokines, interleukin (IL)-6, and interferon (IFN)-*γ* were produced from peripheral blood mononuclear cells (PBMCs) of BD patients, which were stimulated by streptococcal antigen [18], and the serum-antibody titers against *streptococci* were also elevated in BD patients [19]. The peptides of 65 kD of heat shock protein (HSP-65) derived from *streptococci* show considerable homology with those of the human HSP-60 which appeared as counterpart after streptococcal infection [20–22].

Here, an attempt to review on the mucocutaneous manifestations clinically seen in BD patients was done in the connection with the genetic intrinsic and extrinsic triggering factors. We would like to take up a new diagnostic way for BD using self-saliva prick instead of the ordinal “Pathergy test” which seems to be low positive in BD patients.

## 2. Mucocutaneous Involvements

### 2.1. Aphthous Ulceration

**Figure 1:**
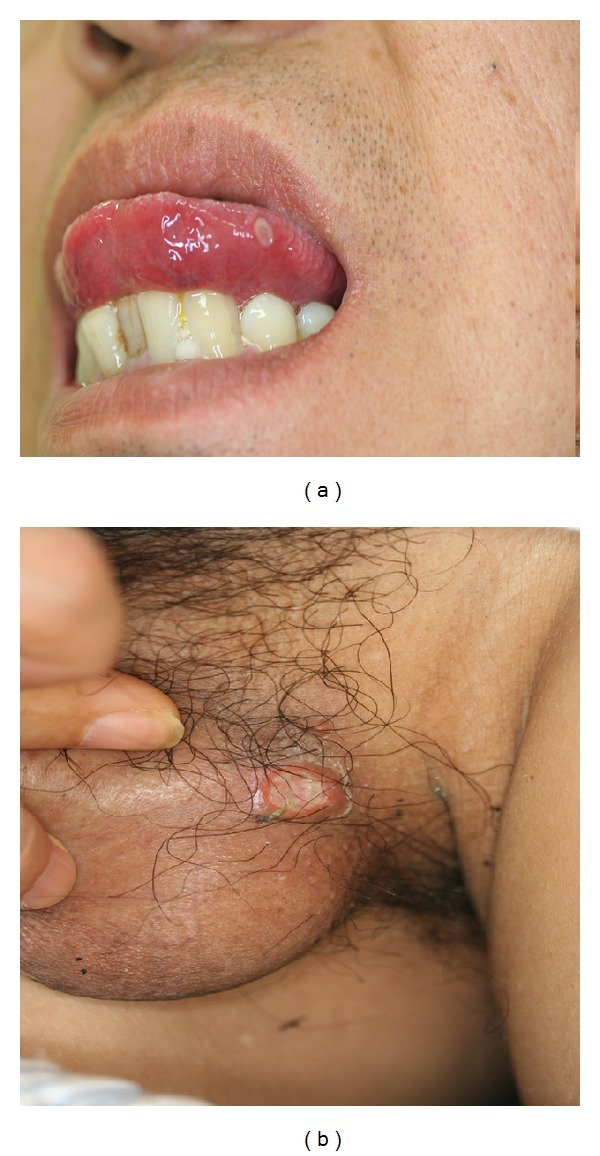
Oral aphtha and genital ulcerations seen in a male BD patient with neuropathy (55 M YY in Table 1). (a) Oral ulcer round and punched-out shaped on the tongue. (b) Genital ulcer shaped like oral ulcer.

**Figure 2:**
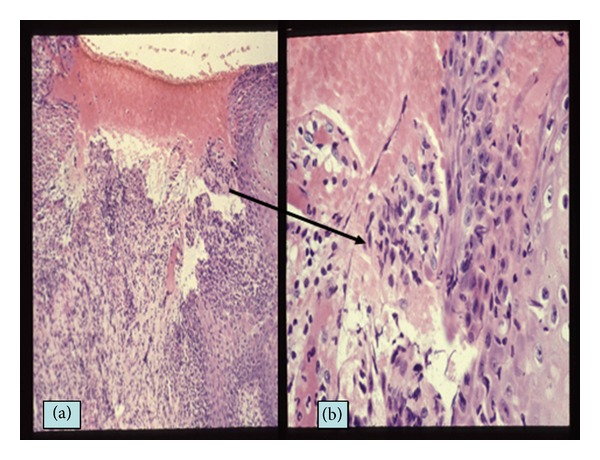
Histology of aphthous ulceration of a BD patient. (a) Aphthous ulcer of the lip defecting the epithelial layer (HE, ×100). (b) Magnified feature of the ulcer edge of the epithelial layer. The epithelial cells are surrounded by inflammatory infiltrates like “Rosetta formation.”

RAS generally starts as an initial symptom in BD patients since their childhood and/or youth and other mucocutaneous symptoms follow after RAS [23–25] (Figures 1(a) and 1(b)). The oral aphthous ulceration punch-out shaped occurs with pain on the tongue, buccal mucosa, gingival, and lip and it continues around a week in BD patients. The clinical features of the oral ulcers is divided as minor, major, herpetiform, and the combined types depending on the lesional size and shapes. Non-BD-RAS is a very common disorder due to trauma, some viral and/or bacterial infections except for patients with BD, and other autoimmune diseases; because it is known that 20% of the general population is affected in the world [26]. On the other hand, nearly 100% of BD patients are associated with RAS as the initial symptom as aphthous ulceration. The biopsy specimen of aphthous ulcer lesion from a BD patient revealed a reaction—like the antibody dependent cell mediated cytotoxicity that the epithelial cells surrounded by neutrophils and lymphoid cells look like leaves falling down from the mucous epithelial layer (Figures 2(a) and 2(b)). These epithelial cells are stained with IgM and HLA-DR and are surrounded by T cells in the immunohistological findings and in addition antistreptococcal antibody was also stained on the cell membrane of the epithelium [15, 27]. However, it is histologically difficult to differentiate aphthous ulceration of BD patient from non-BD-RAS patients.

### 2.2. Genital Ulcer

The clinical features of genital ulceration are generally shaped as similar to oral aphthous ulceration in BD patients (Figure 1(b)) and in young female a genital ulceration suddenly occurs as the initial symptom of BD as Lipschutz ulceration [28], although it was reported to be related to Epstein-Barr viral infection [29]. About more than 50% of BD patients are found to be associated with genital ulceration (female, 55.5%; male, 58.7%); that is, ulcers occur on vulva (66.1%), vaginal mucosa (35.7%), anus (9.6%), cervix (4.1), and groin area (0.8%) in female patients and on the penis (46.5%), scrotum (38.5%), anus (9.2%), and groin area (5.0%) in male patients [23, 25].

### 2.3. EN-Like Eruption

**Figure 3:**
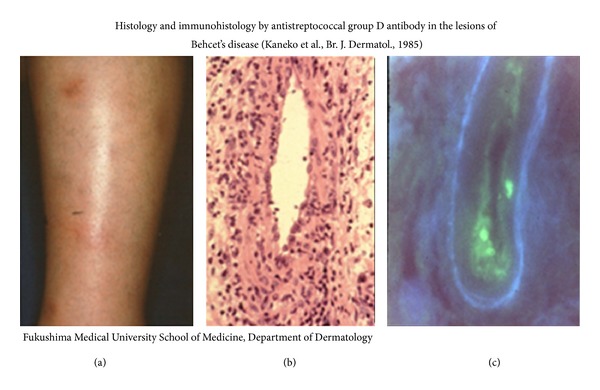
EN-like eruption and the histology and immunohistology. (a) EN-like eruption on the lower legs of a BD patient. (b) Vasculitis infiltrated by lymphoid cells and neutrophils (HE, 400x). (c) Deposits of streptococcal antigen adhering to the vascular wall (direct immunofluorescence, ×400).

More than 50% of BD patients is reported to be associated with EN-like eruption on the legs [23–25, 30], which relatively looks smaller indulation than that of non-BD patients (Figure 3(a)). The histology is generally vascular reaction infiltrated by lymphoid cells, so-called lymphocytic vasculitis, in the dermis and septal panniculitis in the subcutaneous fatty tissue (Figure 3(b)). In acute phase, however, vasculitis surrounded by neutrophils is also able to be recognized. Immunofluorescence technique revealed deposits of IgA, IgM, and complement in the vascular walls and the similar findings can be seen in the reactive site by pathergy test [31–33]. Streptococcal related materials can also be detected in the vascular walls by use of antistreptococcal antibody (Figure 3(c)) [9, 15, 27]. Recently, Cho et al. [34, 35] have demonstrated that IgA and IgM deposited at the lesional vascular walls targeted against human nuclear ribonucleoprotein (hnRNP) A2/B1 of the endothelial cells in BD patients whose serum IgA and IgM also reacted with *S. sanguinis* and HSP-65/60.

### 2.4. Other Cutaneous Disorders

Acne-like eruption due to perifolliculitis repeatedly appears on the upper body of BD patients and subcutaneous thrombophlebitis, so-called “thrombophlebitis migrans,” is suddenly noticed on the lower extremities. Rarely, the follicular lesions may develop to a large ulceration like “pyoderma gangrenosum” on the extremities. Some male BD patients may have a sudden pain and edema of the scrotum due to epididymitis.

## 3. Pathergy Test and Oral Streptococci

The diagnosis of BD is not thought to be difficult for the clinically typical cases who are based on the diagnostic criteria by Japanese and/or International Study Group [24, 36], except for the atypical cases without the main mucocutaneous symptoms including RAS. Pathergy test, which is a nonspecific cutaneous hypersensitive reaction showing around 2 mm pustule 24–48 h after 20 G syringe needle stick, has been thought to be helpful for making a diagnosis of BD for long time, because the phenomenon has been believed as a unique feature for BD. The reactive conditions seem to be varied by the technical method and generally the high positivity is found in Mediterranean and Middle East countries [30]. The reactivity of the “pathergy test” is suggested to be correlated with HLA-B51 in Mediterranean countries [37] and it is one of diagnostic criteria by International Study Group of BD [36]. On the other hand, in the Japanese BD diagnostic criteria “pathergy test” is considered as one of the diagnostic references [23, 24]. However, its positivity in BD patients seems to be chronologically lower to less than 40% of BD patients seen in 2007s, though more than 70% of the patients exhibited positive to the pathergy test in 1970s. The positivity by the test is also different from the prevalence in the countries, as mentioned [38–40]. It is of interest that the surgical cleaning of the forearm before needle prick reduced the prevalence of the “pathergy reaction” [41], suggesting that the positive reaction might be a cutaneous response to some bacteria living on the surface of the skin. In our all cases shown in Tables 1 and 2 none of cutaneous reactions were found 24–48 hours after venipuncture for the clinical examinations using syringe with 22 G needle, because, before the venipuncture, their forearm was cleaned.

**Figure 4:**
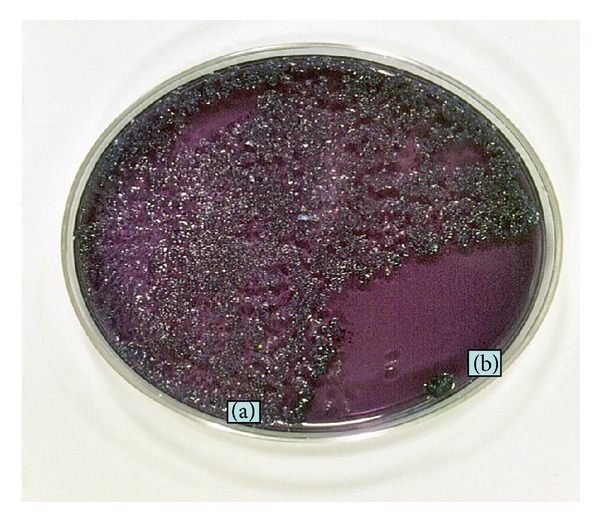
Incubation of saliva of a BD patient using MS (mitis and salivarius) agar in which oral streptococci are selectively grown. (a) Oral streptococci grew from saliva in 5 day. (b) Area of sterilized saliva using syringe micromembrane filter.

**Figure 5:**
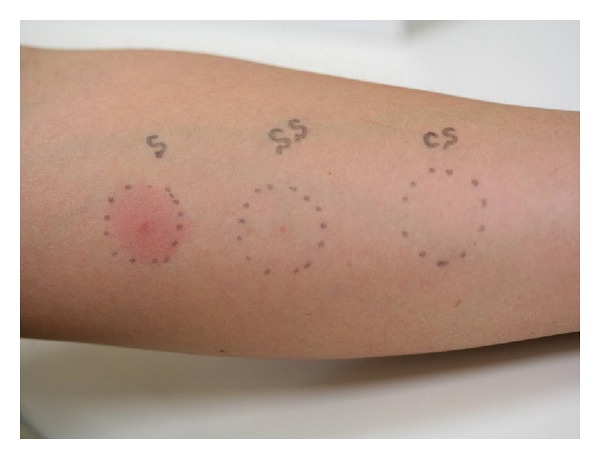
Prick test with self-saliva using Lancetter (33 F AT in Table 1). The skin reactions were observed 48 hours after prick. S: self-saliva; SS: sterilized saliva using syringe-filter with 0.2 *μ*m pores; CS: control saline.

As it is known that many kinds of bacteria are contained in our saliva, we tried to incubate saliva form a BD patient using Mitis-Salivarius (MS) agar which streptococci are selectively grown. The result showed many oral streptococci grew up from pure saliva (Figure 4(a)) and that no bacteria grew from the sterilized saliva by use of a syringe micromembrane filter (Figure 4(b)). Then, instead of conventional “pathergy test,” we tried to prick with self-saliva in which oral bacteria including streptococci are ordinary contained to the forearms of BD patients for diagnosis using a Lancetter with a tiny stick (OY ALGO AB Espoo/Esbo, Sweden) because the patients have hypersensitivity to oral streptococci, as described previously. The results revealed more than 90% of 10 BD patients showed erythematous reaction by stick with self-saliva and that a tiny spot or no reaction was seen by the prick with microfilter-sterilized saliva and control saline (Figure 5, Table 1) [42]. The results also suggest that oral streptococci are playing an important role in the pathogenesis of the RAS of BD patients and that the salivary prick is able to make a differentiation of BD from non-BD disorders. The reaction and severity to self-saliva prick was not related to the possession of the HLA-B51 gene in BD patients (Table 2).

## 4. HLA Genotyping of BD and Streptococcal Infection

HLA-B51 is supposed to be a highly associated genetic marker of BD patients from many different ethnic groups including European, Mediterranean, and Asian people and BD has several unique epidemiologic features from Southern Europe to Japan along “the old silk route” [2, 4, 5, 44]. The appearance of BD lesions is not directly correlated with HLA-B51 in the immunological background of the patients, but it was recently found that HLA-B51-restricted cytotoxic T lymphocytes (CTLs) and *γδ*T cells played some roles in correlation with the stressed target tissues expressing major histocompatibility complex class I-related gene A (MICA) in BD pathogenesis. When the transmembrane-MICA located nearly at the HLA-B51 gene is expressed preferentially on epithelial and endothelial cells by stress, they seem to be the candidates for the HLA-B51-restricted CTLs response and MICA expressed on the stressed epithelium and endothelium which are considered to be the ligand for activating natural killer (NK) cells with NKG2D molecule, *γδ*T cells, and CD8^+^ T cells as CTLs [45]. Regarding NK cell activation, inhibitory CD34/NKG2A and activating CD94/NKG2C molecules are alternatively expressed on NK, CD4^+^CD8^+^ T cells, as indicating an imbalance in cytotoxic activity in BD patients [46], although the function of NK cells is supposed to be down-regulated in the active stage and to be up-regulated in the remission of BD patients [47]. The excessive CD4^+^ T cells activated by inflammatory cytokines including interferon (IFN)-*γ*, IL-12, and IL-23 were altered to Th17 cells and IL-17 which might be released from them in the BD lesions [48].

It is considered that HSP-65/60 derived from microorganism including *S. sanguinis* and from human tissues, which is detected in the oral mucosal and skin lesions of BD patients [20, 21], also becomes a stress-inducible factor in connection with MICA*009 expression. Generally, antigen presenting cells (APCs), which produce IL-12 in correlation with Th1 type immune-reaction, are thought to be activated in BD patients with HLA-B51 in active stage, as indicated by Yasuoka et al. [45]. However, we have obtained the results that PBMCs from BD patients without HLA-B51 gene can be significantly stimulated by *S. sanguinis* antigen in the expression of IL-12p40 mRNA and increasing of protein level in connection with IL-12p70 (70 kDa composed of p35 and p40 subunits) rather than those of the patients with HLA-B51 [51]. It has been suggested that antibacterial host response in T cell type immunity mediated by IL-12 is much stronger in HLA-B51-negative BD patients. The skin response severity by the prick with oral streptococci of self-saliva seemed to be unrelated to the HLA-B51 gene as seen in Table 2.

## 5. Hypersensitivity against *S. sanguinis*

**Figure 6:**
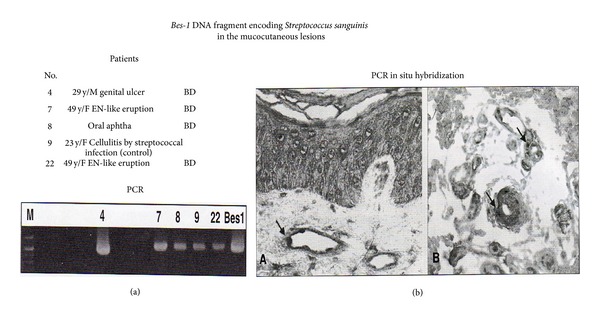
*Bes-1* gene expression in the mucocutaneous lesions of patients with Behcet's disease (BD) [43]. (a) Three of 11 BD patients were positive for* Bes-1* DNA in the lesions including aphthous and genital ulcerations and erythema nodosum (EN)-like eruption by amplified polymerase chain reaction (PCR) using the primers: *Bes-1-1* (5′-TAATAACCCTGACCAAGCCTA-3′) and *Bes-1-2* (5′-CCCTTTCAAAAGTCATAAATC-3′) encoding *S. sanguinis*. (b) In these positive lesions, *Bes-1* DNA was also detected in the cytoplasm of monocytes adhering to the vascular walls and infiltrated around the vessels by PCR in situ hybridization.

Generally, the oral health is impaired in BD patients [8–13], which seems to be associated with the disease severity [10]. Although there are a number of the triggering factors for BD in environmental agent, the predisposition of BD patients may be correlated with streptococcal infection as one of the factors, because the uncommon serotype oral *S*. *sanguinis* is significantly increased in BD patients compared with healthy and disease controls [11–15]. The antibodies against *S. sanguinis* in sera from BD patients showed cross reactivity with the some synthetic peptides of HSP-65 derived from *S*. *sanguinis* [52–54]. The patients show strong delayed type cutaneous hypersensitivity reactions against streptococcal antigens in skin tests [8, 9, 15] and sometimes the BD symptoms were provoked by skin injection of the antigens [16]. Because aphthous ulceration can be also induced by a prick with streptococcal antigen on the oral mucous membrane of a BD patient [9], the appearance of aphthous ulceration is considered to be based on the hypersensitive reaction against *S. sanguinis* which may be traumatically penetrated into the oral membrane of BD patients. Isogai et al. [53] demonstrated that the symptoms mimicking BD appeared in germ-free mice when *S. sanguinis* from BD patients was inoculated into their oral tissue damaged by heat shock and/or mechanical stress. This report suggests that the immunization with *S*. *sanguinis* through the oral membrane route elicits BD-like symptoms in the animal model as seen in BD patients who carry *S. sanguinis* as the pathogenic microorganism in their oral cavity. In order to find polymerase chain reaction (PCR) targeting *Bes-1* gene in BD lesions using 2 distinct primer sets (peptides, 229–243, and 373–385) encoding *S. sanguinis* (serotype KTH-1) which was prepared by Yoshikawa et al. [54], we recognized that *Bes-1* DNA was present in various mucocutaneous lesions including oral and genital ulcerations and EN-like lesions. The PCR in situ hybridization also revealed that *Bes-1* DNA was expressed in the cytoplasm of inflammatory infiltrated monocytes adhering the vascular walls in mucocutaneous lesions (Figure 6) [43]. In contrast, we failed to detect DNAs of HSV-1, HSV-2, cytomegalovirus, human herpes virus (HHV)-6, and HHV-7 in the lesions by PCR [55], although HSV infection has been speculated as etiologically important since the report of Behcet [1]. Interestingly, the amino acid sequence of the peptides of *Bes-1* (229–243 and 373–385) shows more than 60% similarity to the human intraocular ganglion peptide, *Brn-3b* which is a subfamily of POU (pit-Oct Unc) domain factors containing *Brn-3a* and *Brn-3c* [56]. The peptide of *Bes-1* (229–243) was also found to be correlated with the peptide of HSP-60 (336–351) [54]. Recently, it has been found that the peptide of *Bes-1* (337–385) stimulated PBMCs of BD patients which produced IFN-*γ* and IL-12, though the cellular proliferation of the stimulated PBMCs was not observed [57]. These results suggest that *Bes-1* derived from oral *S. sanguinis* might be an inducer for the possible retinal and neural involvement in BD patients.

## 6. HSPs and BD Pathogenesis

Antibodies against the HSP peptides derived from bacteria including *S. sanguinis* are found in aphthous ulceration and serum of BD patients [58], though HSP specific antibodies and T cells are considered to play a complicated role in the pathogenesis of human autoimmune diseases [59]. It is speculated that HSPs trigger both innate and adaptive immune mechanisms in BD. On the other hand, the therapeutic approaches involving HSP immunomodulation may be available as “oral toleration” using the peptide of HSP (336–351) linked to recombinant cholera toxin B for BD patients with advanced uveitis, as demonstrated by Stanford et al. [60]. In order to understand the suppressive mechanisms of the cytokine production in PBMCs from active BD patients, we tried to find the binding sites of the peptides on monocytes by cDNA chips (Gene Chip; Human Genome) using NOMO-1 cells (human macrophage cell line) activated by *S. sanguinis* antigen and they were incubated with the peptides. It was found that although the expression of IL-8, IL-16, IL-13R, and IL-17R was decreased after incubation with HSP-65 peptides (LO1 and UK), respectively, LO2 (480–499) did not decrease IL-8 production. CD58 (lymphocyte function-associated antigen-3) molecule and/or FK506 binding protein were highly expressed on the cell membrane by LO1 (249–264) and UK (311–326) [61, 62].

## 7. Toll-Like Receptor (TLR) Expression in the Innate Immunity

Regarding the recognition system for the microorganism antigens in humans, 10 numbers of TLR family are supposed to act as innate immune receptors by binding of particular structures present on bacteria, viruses, fungi, and so forth [63]. Although generally TLRs are weakly detectable in various human tissues with varying levels, the TLR expression of the organs involved in immune response and exposed to environment is found to be significantly stronger [64]. TLR-3 [ds RNA] and TLR-6 [mycoplasma, staphylococci, etc.] are also reported to be enhanced in expression on neutrophils and monocytes of BD patients, when stimulated by HSP-60 and *S. sanguinis *antigen [65]. In the oral ulcer lesion, expression of TLR-9 [unmethylated CpG DNA, bacteria, and virus] has been found recently [66]. These findings suggest that innate immune system contributes the acquisition of hypersensitivity against oral *S. sanguinis* as the extrinsic factor in the pathogenesis of BD.

## 8. Oral Aphthous Ulceration and Systemic Symptoms

**Figure 7:**
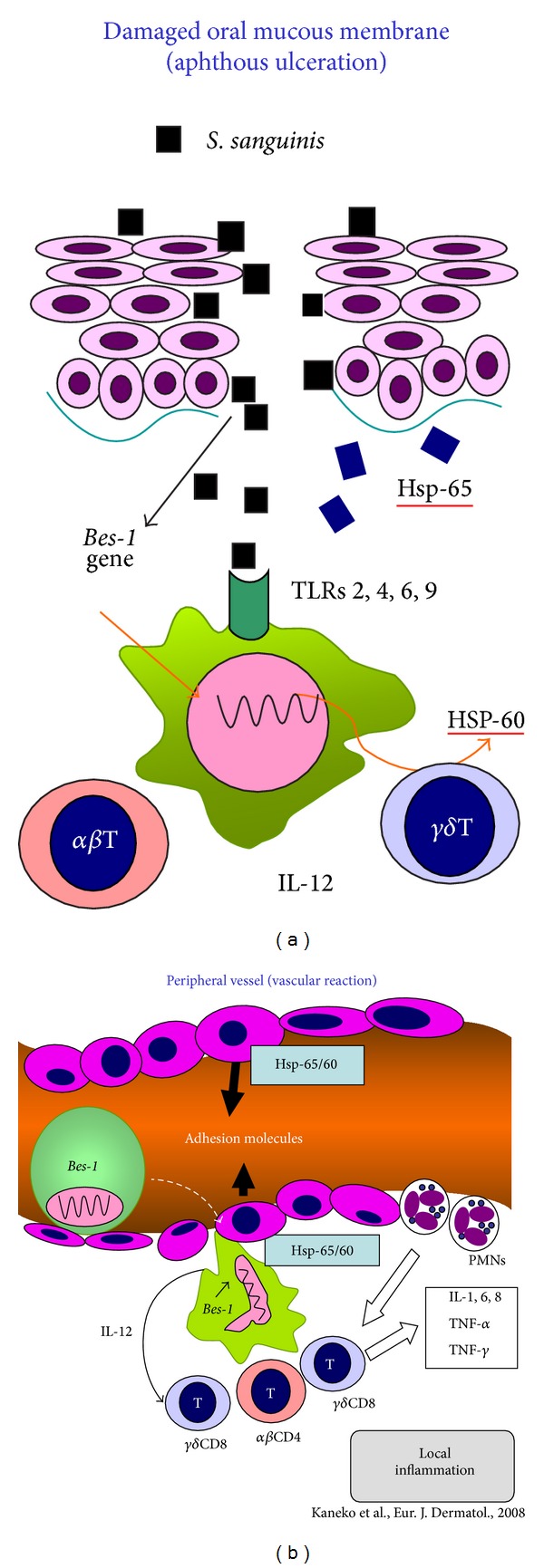
Hypothesis of the mechanisms in the appearance of various lesions of BD patients [49, 50]. (a) The antigen presenting cells (APCs) (macrophages and/or dendritic cells) immunized by *S. sanguinis* agents though TLRs in the oral cavity might be carried to the peripheral regions. (b) If the APCs in the blood flow adhered to the impaired and/or MICA and adhesion molecules expressed endothelial cells of vascular wall, the immunological reaction might be appeared as BD lesion.

BD symptoms are characterized by vascular involvements showing swollen endothelial cells of the microarteries infiltrated by inflammatory monocytes and a few neutrophils histologically, as so-called “vascular reaction” seen in EN-like eruption and other lesions [15, 31, 67, 68]. The strong hypersensitivity reaction against *S. sanguinis* agents [8, 9, 15, 16, 18] which might be gained by antigen present cells (APCs) through the innate immune mechanism can be suspected as the extrinsic triggering factor in the pathogenesis of BD. In the treatment by antibiotics for the involvement of oral *S. sanguinis*, especially minocycline, which not only reduces the growth of streptococci but also suppresses IL-1*β* and IL-6 production from T cells inflamed, was clinically effective for aphthous ulceration, acne-like eruption and EN-like lesion in BD patients [9]. Other study also showed that combination therapy, colchicine and benzathine penicillin, was effective to suppress BD symptoms compared to colchicine monotherapy [69, 70]. Although Kaneko et al. [49] and others [5–7] have already reviewed on the role of infectious agents in BD pathogenesis, we also dare to propose the hypothesis that after *Bes-1* gene taken in the cytoplasm of APCs through the TLRs in the oral cavity, the APCs, which are expressing the streptococcal antigen, produce HSP-65/60, as demonstrated by Deniz et al. [58]. If these APCs are curried in the blood flow to the impaired and/or MICA expressed endothelium of the vessels in correlation with HSP-65/60, VEGF, adhesion molecules, and so forth, BD lesions might be induced by the “vascular reaction” and/or “lymphocytic vasculitis” as the immunological reaction by the APCs expressing *S. sanguinis* antigen. Then, the relationship between oral ulceration and the systemic symptoms might be considered as illustrated in Figure 7. From the viewpoint, it is considerable that the positivity of the prick with self-saliva is high for BD patients [49, 50, 62]. So, we would like to propose a new diagnostic way for BD and differentiation from non-BD patients and/or non-BD RAS patients.

## Figures and Tables

**Table 1:** Self-salivary prick test in patients with aphthous ulceration and controls.

Patients	Age, sex (initials)	Prick test	Small pustule	SS-prick	CS-prick
		(after 48 h)			
Neuro BD	55 M YY	11 × 15 mm	+	nd	−

Incomplete	33 F AT*	22 × 22	−	−	−
	26 F MN*	10 × 10	+	+ dot	−
	27 M TG	11 × 12	+	+ dot	−
	47 M YT	10 × 13	+	+ dot	−
	36 F AN*	5 × 10	−	nd	−
	46 M KH	10 × 10	−	−	−
	17 F YT	5 × 5	−	−	−

Complete	23 M OO*	10 × 10	+	−	−

Recurrent aphthous stomatitis (non-BD RAS)	24 F YN	8 × 10 mm	−	−	−
	28 F YS	8 × 4	−	−	−
	32 F YN	−	−	nd	−
	29 M ON	−	−	nd	−
	28 F MS	3 × 5	−	−	−

Disease controls non-BD EN	39 F KY	−	−	nd	−
	61 F MF	−	−	—	−

Viral aphthosis (3)		−	−	nd	−
Healthy controls (6)		−	−	−	−

BD: Behcet's disease; EN: erythema nodosum; F: female; M: male; dot: small spot; +: positive; −:negative. S-prick: prick with self-saliva; SS-prick: prick with sterilized self-saliva; CS: prick with saline; nd: not done. The clinical type of BD is followed by the Japanese BD classification. *Same cases in Table 2.

**Table 2:** Self-salivary prick test in BD patients with or without HLA-B51.

Type of BD (Japanese classification)	Patients (initials)	Prick test (mm)			HLA-B51
		S	SS	CS	
Complete type	23 M OO*	10	—	−	+ (B51)

Incomplete type	40 M HG	10	7	−	+ (B51, 01, 01)
	31 F MA	30	7	−	+ (B51, 40)
	42 F MK	7	4	−	+ (B51, 46, DR4, 8)
	34 F MY	26	5	−	− (B35, 48)
	36 F AN*	10	−	−	− (B44, 03, 01)
	33 F YK	10	−	−	−
	37 F AT*	22	−	−	− (B40, 48)
	30 F MN*	10	−	−	−
	35 M YI	10	nd	−	− (B15, 35)
	37 F HT	4	−	−	− (B40, 44)
	36 M MK	4	3	−	− (B35, 44)
	35 M KF	7	2	−	− (B46, 54)
	35 F YO	14	−	−	− (B07, 02, 01)

F: female; M: male; S: self-saliva; SS: filtered sterilized saliva; CS: control saline; +: positive; −: negative; nd: not done. *Same cases in Table 1.

## References

[1] Behcet H (1937). Uber rezidivierende, aphthous durch ein Virus verursachte Geschwuere am Mund, am Auge und an den Genitalien. *Dermatologische Wochenschrift*.

[2] Altenburg A, Papoutsis N, Orawa H, Martus P, Krause L, Zouboulis CC (2006). Epidemiology and clinical manifestations of Adamantiades-Behcet disease in Germany—current pathogenetic concepts and therapeutic possibilities. *Journal of the German Society of Dermatology*.

[3] Alpsoy E, Zouboulis C, Ehrlich GE (2007). Mucocutaneous lesions of Behcet’s disease. *Yonsei Medical Journal*.

[4] Ohno S, Onguchi M, Hirose S, Matsuda H, Wakisaka A, Aizawa M (1982). Close association of HLA-Bw51 with Behcet’s disease. *Archives of Ophthalmology*.

[5] Zouboulis CC, May T (2003). Pathogenesis of Adamantiades-Behcet’s disease. *Medical Microbiology and Immunology*.

[6] Karaycian A, Zouboulis CC (2007). An update on Behcet’s disease. *Journal of the European Academy of Dermatology and Venereology*.

[7] Krause I, Weinberger A (2008). Behcet’s disease. *Current Opinion in Rheumatology*.

[8] Kaneko F, Kaneda T, Ohnishi O (1978). Infection allergy in Behcet’s disease. *Japanese Journal of Allergology*.

[9] Kaneko F, Oyama N, Nishibu A (1997). Streptococcal infection in the pathogenesis of Behcet’s disease and clinical effects of minocycline on the disease symptoms. *Yonsei Medical Journal*.

[10] Mumcu G, Ergun T, Inanc N (2004). Oral health is impaired in Behcet's disease and is associated with disease severity. *Rheumatology*.

[11] Galeone M, Colucci R, D’Erme AM, Moretti S, Lotti T (2012). Potential infectious etiology of Behcet’s disease. *Pathology Research International*.

[12] Yokota K, Hayashi S, Araki Y (1995). Characterization of *Streptococcus sanguis* isolated from patients with Behcet’s disease. *Microbiology and Immunology*.

[13] Isogai E, Ohno S, Takashi K (1990). Close association of *Streptococcus sanguis* uncommon serotypes with Behcet’s disease. *Bifidobacteria Microflora*.

[14] Isogai E, Ohno S, Kotake S (1990). Chemiluminescence of neutrophils from patients with Behcet’s disease and its correlation with an increased proportion of uncommon serotypes of *Streptococcus sanguis* in the oral flora. *Archives of Oral Biology*.

[15] Kaneko F, Takahashi Y, Muramatsu Y, Miura Y (1985). Immunological studies on aphthous ulcer and erythema nodosum-like eruptions in Behcet’s disease. *The British Journal of Dermatology*.

[16] Mizushima Y, Matsuda T, Hoshi K, Ohno S (1988). Induction of Behcet’s disease symptoms after dental treatment and streptococcal antigen skin test. *Journal of Rheumatology*.

[17] Graykowski EA, Barile MF, Lee WB, Stanley HR (1966). Recurrent aphthous stomatitis. Clinical, therapeutic, histopathologic, and hypersensitivity aspects. *The Journal of the American Medical Association*.

[18] Hirohata S, Oka H, Mizushima Y (1992). Streptococcal-related antigens stimulate production of IL6 and interferon-*γ* by T cells from patients with Behcet’s disease. *Cellular Immunology*.

[19] Yokota K, Hayashi S, Fuji N (1992). Antibody response to oral Streptococci in Behcet’s disease. *Microbiology and Immunology*.

[20] Lehner T (1997). The role of heat shock protein, microbial and auto-immune agents in the etiology of Behcet’s disease. *International Reviews of Immunology*.

[21] Kaneko S, Suzuki N, Yamashita N (1997). Characterization of T cells specific for an epitope of human 60-kD heat shock protein (hsp) in patients with Behcet’s disease (BD) in Japan. *Clinical and Experimental Immunology*.

[22] Kibaroglu A, Eksioglu-Demiralp E, Akoglu T, Direskeneli H (2004). T and NK cell subset changes with microbial extracts and human HSP60-derived peptides in Behcet’s disease. *Clinical and Experimental Rheumatology*.

[23] Sakane T, Takeno M, Suzuki N, Inaba G (1999). Behcet’s disease. *The New England Journal of Medicine*.

[24] Suzuki-Krokawa M, Suzuki N (2004). Behcet’s disease. *Clinical and Experimental Medicine*.

[25] Bang D, Yoon KH, Chung HG, Choi EH, Lee ES, Lee S (1997). Epidemiological and clinical features of Behcet’s disease in Korea. *Yonsei Medical Journal*.

[26] Ship JA (1996). Recurrent aphthous stomatitis. An update. *Oral Surgery, Oral Medicine, Oral Pathology, Oral Radiology, and Endodontics*.

[27] Kaneko F, Ueki H, Yaoita H (1989). Behcet’s disease. *A Color Atlas of Dermato-Immunohistology*.

[28] Lipschutz B, Jadassohn J (1927). Ulcer vulvae acutum. *Handbuch der Haut und Geschl*.

[29] Lampert A, Assier-Bonnet H, Chevallier B, Clerici T, Saiag P (1996). Lipschutz’s genital ulceration: a manifestation of Epstein-Barr virus primary infection. *The British Journal of Dermatology*.

[30] Lee S, Bang D, Lee ES (2003). Behcet’s disease. *The 3rd List of Publication on Behcet’s Disease. 1995–2002*.

[31] Haim S, Sobel JD, Friedman Birnbaum R, Lichtig C (1976). Histological and direct immunofluorescence study of cutaneous hyperreactivity in Behcet’s disease. *The British Journal of Dermatology*.

[32] Jorizzo JL, Abernethy JL, White WL (1995). Mucocutaneous criteria for the diagnosis of Behcet’s disease: an analysis of clinicopathologic data from multiple international centers. *Journal of the American Academy of Dermatology*.

[33] Serhat Inaloz H, Evereklioglu C, Unal B, Kirtak N, Eralp A, Inaloz SS (2004). The significance of immunohistochemistry in the skin pathergy reaction of patients with Behcet’s syndrome. *Journal of the European Academy of Dermatology and Venereology*.

[34] Cho S, Zheng Z, Cho S (2013). Both the sera of patients with Behcet’s disease and Streptococcus sanguis stimulate membrane expression of hnRNP A2/B1 in endothelial cells. *Scandinavian Journal of Rheumatology*.

[35] Cho SB, Zheng Z, Ahn KJ (2013). Serum IgA reactivity against GroEL of *Streptococcus sanguinis* and human heterogeneous nuclear ribonucleoprotein A2/B1 in patients with Behcet’s disease. *The British Journal of Dermatology*.

[36] International Study Group for Behcet’s Disease (1990). Criteria for diagnosis of Behcet’s disease. *The Lancet*.

[37] Yazici H, Tuzun Y, Pazarli H, Yalcin B, Yurdakul S, Muftuoglu A (1980). The combined use of HLA-B5 and the pathergy test as diagnostic markers of Behcet’s disease in Turkey. *Journal of Rheumatology*.

[38] Davatchi F, Chams-Davatchi C, Shahram F (2007). Pathergy test in Behcet’s disease: change in incidence over the time. *APLAR Journal of Rheumatology*.

[39] Davies PG, Fordham JN, Kirwan JR, Barnes CG, Dinning WJ (1984). The pathergy test and Behcet’s syndrome in Britain. *Annals of the Rheumatic Diseases*.

[40] Friedman-Birnbaum R, Bergman R, Aizen E (1990). Sensitivity and specificity of pathergy test results in Israeli patients with Behcet’s disease. *Cutis*.

[41] Fresko I, Yazici H, Bayramicli M, Yurdakul S, Mat C (1993). Effect of surgical cleaning of the skin on the pathergy phenomenon in Behcet’s syndrome. *Annals of the Rheumatic Diseases*.

[42] Togashi A, Saito S, Kaneko F, Nakamura K, Oyama N (2011). Skin prick test with self-saliva in patients with oral aphthoses: a new diagnostic pathergy for behcet’s disease and recurrent aphthosis. *Inflammation and Allergy—Drug Targets*.

[43] Tojo M, Yanagihori H, Zheng X (2003). Bes-1 DNA fragment encoding streptococcal antigen in skin lesions from patients with Behcet’s disease. *Journal of Applied Research*.

[44] Mizuki N, Inoko H, Ohno S, Lee S, Bang D, Lee E-S, Sohn S (2001). Molecular genetics (HLA) of Behcet’s disease. *Behcet’s Disease—A Guide to Its Clinical Understanding*.

[45] Yasuoka H, Okazaki Y, Kawakami Y (2004). Autoreactive CD8+ cytotoxic T lymphocytes to major histocompatibility complex class I chain-related gene A in patients with Behcet’s disease. *Arthritis and Rheumatism*.

[46] Seo J, Park JS, Nam JH (2007). Association of CD94/NKG2A, CD94/NKG2C, and its ligand HLA-E polymorphisms with Behcet’s disease. *Tissue Antigens*.

[47] Kaneko F, Takahashi Y, Muramatsu R (1985). Natural killer cell numbers and function in peripheral lymphoid cells in Behcet’s disease. *The British Journal of Dermatology*.

[48] Shimizu J, Takai K, Fujiwara N (2012). Excessive CD4+ T cells co-expressing interleukin-17 and interferon-*γ* in patients with Behcet’s disease. *Clinical and Experimental Immunology*.

[49] Kaneko F, Oyama N, Yanagihori H, Isogai E, Yokota K, Oguma K (2008). The role of streptococcal hypersensitivity in the pathogenesis of Behcet’s disease. *European Journal of Dermatology*.

[50] Kaneko F, Togashi A, Saito S (2011). Behcet’s disease (Adamantiades-Behcet’s disease). *Clinical and Developmental Immunology*.

[51] Yanagihori H, Oyama N, Nakamura K, Mizuki N, Oguma K, Kaneko F (2006). Role of IL-12B promoter polymorphism in Adamantiades-Behcet’s disease susceptibility: an involvement of Th1 immunoreactivity against *Streptococcus sanguinis* antigen. *Journal of Investigative Dermatology*.

[52] Isogai E, Isogai H, Kotake S (2002). Antibody cross reactivity from sera of patients with behcet’s disease with synthetic peptides that have homologies with proteins from *Streptococcus sanguis*. *Journal of Applied Research*.

[53] Isogai E, Isogai H, Kotake S, Ohno S, Kimura K, Oguma K (2003). Role of *Streptococcus sanguis* and traumatic factors in Behcet’s disease. *Journal of Applied Research*.

[54] Yoshikawa K, Kotake S, Kubota T, Kimura K, Isogai E, Fujii N (1998). Cloning and sequencing of BES-1 gene encoding the immunogenic antigen of *Streptococcus sanguis* KTH-1 isolated from the patients with Behcet’s disease. *Zentralblatt fur Bakteriologie*.

[55] Tojo M, Zheng X, Yanagihori H (2003). Detection of herpes virus genomes in skin lesions from patients with Behcet’s disease and other related inflammatory diseases. *Acta Dermato-Venereologica*.

[56] Xiang M, Zhou L, Peng Y, Eddy RL, Shows TB, Nathans J (1993). Brn-3b: a POU domain gene expressed in a subset of retinal ganglion cells. *Neuron*.

[57] Kulaber A, Tugal-Tutkun I, Yentür SP (2007). Pro-inflammatory cellular immune response in Behcet’s disease. *Rheumatology International*.

[58] Deniz E, Guc U, Buyukbabani N, Gul A (2010). HSP 60 expression in recurrent oral ulcerations of Behet’s disease. *Oral Surgery, Oral Medicine, Oral Pathology, Oral Radiology and Endodontology*.

[59] Zugel U, Kaufman HE (1999). Role of heat shock proteins in protection from and pathogenesis of infectious diseases. *Clinical Microbiology Reviews*.

[60] Stanford M, Whittall T, Bergmeier LA (2004). Oral tolerization with peptide 336-351 linked to cholera toxin B subunit in preventing relapses of uveitis in Behcet’s disease. *Clinical and Experimental Immunology*.

[61] Oguma K, Shin R, Yokota K (2008 (Japanese)). Studies on immunological responses by bacterial antigens in Behcet’s disease. *Report of the Research Group for Behcet’s Disesae Organized by the Japanese Ministry of Health, Lavour and Welfare 2006-2007*.

[62] Kaneko F, Togashi A, Nomura E (2012). Role of heat shock protein derived from *Streptococcus sanguinis* in Behcet’s disease. *Journal of Medical Microbiology and Diagnosis*.

[63] Zarember KA, Godowski PJ (2002). Tissue expression of human toll-like receptors and differential regulation of toll-like receptor mRNAs in leukocytes in response to microbes, their products, and cytokines. *Journal of Immunology*.

[64] Hornung V, Rothenfusser S, Britsch S (2002). Quantitative expression of toll-like receptor 1–10 mRNA in cellular subsets of human peripheral blood mononuclear cells and sensitivity to CpG oligodeoxynucleotides. *Journal of Immunology*.

[65] Yavuz S, Elbir Y, Tulunay A, Eksioglu-Demiralp E, Direskeneli H (2008). Differential expression of toll-like receptor 6 on granulocytes and monocytes implicates the role of microorganisms in Behcet’s disease etiopathogenesis. *Rheumatology International*.

[66] Durranni O, Wallace GR, Hamburger J (2004). Toll-like receptors (TLRs) expression in oral ulcer biopsies from Behcet’s disease (BD) patients: a role for the innate immune system in BD. *Clinical and Experimental Rheumatology*.

[67] Yi SW, Kim EH, Kang HY, Kim YC, Lee ES (2007). Erythema nodosum: clinicopathologic correlations and their use in differential diagnosis. *Yonsei Medical Journal*.

[68] Iikunur T, Pabuccuoglu U, Akin C, Lebe B, Gunes AT (2006). Histopathologic and direct immunofluorescence findings of the papulopustular lesions in Behcet’s disesase. *European Journal of Dermatology*.

[69] Çalgüneri M, Ertenli I, Kiraz S, Erman M, Çelik I (1996). Effect of prophylactic benzathine penicillin on mucocutaneous symptoms of Behcet’s disease. *Dermatology*.

[70] Mumcu G, Inanc N, Yavuz S, Direskeneli H (2007). The role of infectious agents in the pathogenesis, clinical manifestations and treatment strategies in Behcet’s disease. *Clinical and Experimental Rheumatology*.

